# Prevalence of invasive aspergillosis in suspected pulmonary tuberculosis at a referral tuberculosis hospital in Shandong, China

**DOI:** 10.1017/S095026882000268X

**Published:** 2020-11-05

**Authors:** Jun-Li Wang, Xiao-Lin Zhou, Chao Han, Mao-Shui Wang, Hua Hu

**Affiliations:** 1 Department of Lab Medicine, The Affiliated Hospital of Youjiang Medical University for Nationalities, Baise, China; 2 Department of Lab Medicine, Shandong Provincial Chest Hospital, Shandong University, Jinan, Shandong, China; 3 Department of Geriatrics, Shandong Mental Health Center, Jinan, Shandong, China; 4 Department of Pulmonary and Critical Care Medicine, Shandong Provincial Chest Hospital, Shandong University, Jinan, China

**Keywords:** Aspergillus, cavity, prevalence, tuberculosis

## Abstract

Although the progression of invasive aspergillosis (IA) shares some risk factors in the development of active pulmonary tuberculosis (PTB), however, the prevalence of IA in suspected PTB remains unclear. During a period of 1 year (from January 2016 to December 2016), consecutive patients with suspected PTB were included in a referral TB hospital. Data, including demographic information and underlying diseases, were collected from medical records. PTB were all confirmed by mycobacterial culture (Lowenstein–Jensen medium). IA were diagnosed as proven or probable according to the criteria of the 2008 EORTC/MSG definitions. A descriptive analysis was performed to estimate the corresponding prevalence. During the study year, 1507 patients have a positive mycobacterial culture, with a mean age of 45.6 (s.d. 19.9) years old and a female:male ratio of 1:4. Among the 82 patients with non-tuberculous mycobacterial diseases, two patients (2.44%, 95% CI 0.67–8.46%) were diagnosed as IA (one proven and one probable); two probable IA patients (0.15%, 95% CI 0.04–0.55%) were diagnosed in PTB patients (*n* = 1315), and all were retreatment cases. In addition, all four IA patients (100%) exhibited cavities in both lobes on radiograph. In China, the prevalence of IA is low in active PTB patients. However, when high-risk factors for IA are encountered in PTB patients, further investigations are required and empirically treatment for IA might be warranted.

## Introduction

Currently, tuberculosis (TB) remains a serious public health threat. According to WHO (2019), an estimated 10.0 million people fell ill with TB, and an estimated 1.4 million TB deaths occurred worldwide in 2018 [1]. As known, all factors associated with immune status may have an effect on the development of TB. Until now, several factors, such as HIV infection, undernutrition, diabetes, smoking, alcohol consumption, transplant recipients and malignancy [2–8], have been identified in the development of pulmonary tuberculosis (PTB). Similarly, immune-compromised status is also a major factor preceding aspergillosis, and the above-mentioned risk factors are also shared in the development of invasive aspergillosis (IA), such as HIV infection [9], transplant recipients [10], malignancy [11] and smoking [12], which has been investigated in various studies. On the other hand, pulmonary cavity usually is a result of a previous TB, and these residual cavities become infected with Aspergillus and pulmonary aspergillosis then develops following inhalation of airborne fungal spores [13]. This suggests that retreatment TB cases are at a high risk of developing IA.

IA is a common infection in immune-compromised patients. Despite advances in therapy, IA remains a serious and fatal opportunistic infection. The prevalence of aspergillosis complicating TB has been reported in several studies from different countries, and the results demonstrated that the prevalence varied widely, and was up to 25% in active TB cases [14–18]. Additionally, in a recent meta-analysis, a pooled rate of Aspergillus coinfection among patients with pulmonary TB was reported at 15.4% (95% CI 11.4–20.5) in Asia and Africa [19]. This variance may be explained by the difference in the diagnostic criteria and geographical distribution of IA. However, in China, the prevalence of IA in patients with suspected PTB remains unclear. Hence, in this retrospective study, we aimed to evaluate the prevalence of IA in suspected PTB.

## Materials and methods

The study protocol was approved by the ethical committee of the Shandong Provincial Chest Hospital (SPCH). Written informed consent was waived by the ethical committee of SPCH because of the retrospective study design and an absence of personal information. The investigations were carried out in accordance with the Declaration of Helsinki.

During a period of 1 year (from January 2016 to December 2016), consecutive patients with suspected PTB were included in SPCH. Data, including demographic information and underlying diseases, were collected from medical records. PTB were all confirmed by mycobacterial culture (Lowenstein–Jensen medium). Non-tuberculous mycobacterial (NTM) disease was diagnosed according to American Thoracic Society criteria [20]. Retreatment TB was defined as a new TB diagnosis in a patient who had previously completed TB treatment.

In suspected IA cases, bronchoscopic alveolar lavage fluid (BALF) and serum samples were collected and sent for galactomannan (GM) assay (DNK Ltd, Tianjin, China). Computed tomography-guided percutaneous transthoracic needle biopsies were performed in suspected IA cases and then processed for pathological examination. BALF, sputum and tissues were processed for fungus culture. Two samples were cultured on two Sabouraud agar plates separately and incubated at 37 and 45°C for up to 5 days. The isolated fungus was identified by its colony characteristics and morphological features.

IA were diagnosed as proven or probable according to the criteria of the 2008 EORTC/MSG definitions [21]. Briefly, proven IA was defined by histology showing hyphal tissue invasion (Gomori methenamine-silver, with hyphal walls staining dark) and microbiological proof of Aspergillus infection. Probable IA was established according to radiologic findings plus a positive BALF culture, or a positive GM assay (ELISA method) on BALF (⩾0.90 μg/l) or serum (⩾0.75 μg/l).

All analyses were performed using SPSS version 16.0 (SPSS, Chicago, IL, USA). Due to small numbers of IA patients, a descriptive analysis was performed. The continuous data are presented as the mean ± s.d. and categorical data as frequencies (percentage). The prevalence was estimated and the corresponding 95% confidence interval (CI) was also calculated.

## Results

**Fig. 1.**
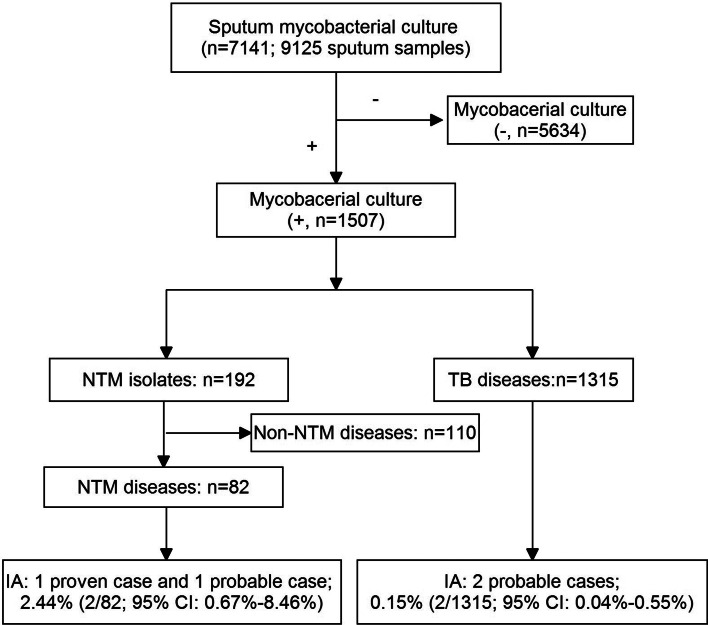
A flow chart of the patients included in the study.

During the study year, 9125 sputum collected from 7141 suspected PTB patients were sent for mycobacterial culture (Fig. 1). Of them, 1507 patients had a positive mycobacterial culture, with a mean age of 45.6 (s.d. 19.9) years old and a female:male ratio of 1:4 (female, *n* = 487, 41.2 (s.d. 16.7) years; male, *n* = 47.7 (s.d. 19.7) years). Subsequently, NTM diseases were diagnosed in 82 (5.4%) patients, and TB in 1315 (87.3%) patients, the remaining 110 (7.3%) patients were excluded for the final diagnosis of NTM diseases could not be established. Of the 1315 PTB patients, 232 (17.6%) were retreatment cases. HIV status was examined in 1499 suspected PTB patients (mycobacterial culture, +), and all of them were HIV-negative.

A total of four IA patients were identified in this study, including one proven case and three probable cases. The proven case was confirmed using tissue culture, probable cases were established according to radiologic findings plus a positive BAL culture in one patient, and a positive GM on BAL in two patients. The four IA patients all exhibited cavities in both lobes on radiograph. Additionally, all IA patients were administrated with voriconazole orally (200 mg twice a day) for 6 months.

Among the patients with NTM disease, two patients were diagnosed as IA (one proven and one probable). Therefore, a prevalence of 2.44% (95% CI 0.67–8.46%) of IA in NTM diseases was estimated. Likewise, two probable IA patients were diagnosed in culture-confirmed PTB patients, and a prevalence of 0.15% (95% CI 0.04–0.55%) of IA in PTB was then estimated. Interestingly, the two IA complicating TB patients were all retreatment cases. Therefore, among retreatment TB cases, the prevalence of IA was 0.86% (95% CI 0.24–3.09%).

## Discussion

To our knowledge, this is the first report to systematically evaluate the prevalence of IA in active PTB in China. In the study, a low prevalence was observed, and these cases were all associated with common risk factors, such as NTM disease or retreatment TB.

Interestingly, raised Aspergillus-specific antibodies were observed in TB patients when administered with anti-TB therapy [22]. For example, in a study by Chu *et al*. [20], a serological assay was used to evaluate the prevalence of Aspergillus infection, with a prevalence of up to 25% in patients with old TB or bronchiectasis. Although several different methods for antibody against Aspergillus were used to define the infection and have different sensitivities [23], this phenomenon suggests that Aspergillus infection was common to complicate active PTB. Additionally, a high prevalence of Aspergillus infection was estimated at 12–22% in African TB patients with cavities [24]. In general, these data mentioned above mean a possible association between IA and TB. As known, the diagnostic criteria for the Aspergillus infection are arbitrary and based on expert opinion [25], and therefore varied between studies. Hence, standardised and validated tests, such as tissue culture and pathological examinations, are recommended.

However, the prevalence of IA was low in active TB cases. For example, in a study from Iran, IA was observed as co-infection with *Mycobacterium tuberculosis* in 3.72% of suspected PTB cases [16]. Oladele RO *et al*. found 8.7% of patients with smear-negative TB and treatment failure can establish an alternative diagnosis of chronic pulmonary aspergillosis (CPA) based on serological assay, chest X-ray, culture and symptoms [26]. In addition, a recent report from China showed that IA complicating active TB occurs in a minority (about 5%) of paediatric patients with chronic granulomatous disease [27]. In our study, a lower prevalence of IA in active PTB patients was observed. Geographical distribution may be responsible for the difference in the prevalence of IA between different studies [19]. As reported, the prevalence of Aspergillus infection showed a considerable geographical variation, and China had an intermediate prevalence of CPA in previous TB patients [28, 29].

IA usually complicates several underlying lung diseases, such as previous PTB, NTM infection, COPD and bronchiectasis [29]. The existing data have shown that a previous PTB is associated with the occurrence of CPA [13]. Furthermore, a high prevalence of Aspergillus sensitisation was reported in PTB-related fibrocavitary disease [30]. In high TB burden countries, TB is usually the most common primary underlying condition in the development of CPA [31]. However, compared with other lung conditions, it has a relatively good prognosis. Our findings are consistent with these features. Four IA patients were diagnosed, two of them were retreatment TB cases, another two of them had NTM disease, and all of them exhibited cavities on radiograph. As reported, the Aspergillus infection was frequently seen in those with chest radiography cavitation than without it [13]. This suggests that PTB patients should have chest radiography and that those with cavities should be monitored for Aspergillus to facilitate early treatment.

This study has several limitations. First, due to the economic and geographical condition of the area (Shandong) in China, our data may be further used to assess the situation of other areas in eastern China. However, when generalised to other areas, such as most areas in western China, our data may have limited clinical value. Second, due to the low incidence of the invasive forms of Aspergillus infection, a small number of patients included implies low statistical power to estimate the prevalence. Third, this is an institutional study, so the results may have a selection bias. Fourth, as known, most clinical and radiological characteristics of PTB overlapped with IA. Therefore, although all TB cases were culture-confirmed, some patients complicating IA may be diagnosed as having only TB.

## Conclusions

Our data demonstrated that the prevalence of IA is low in active PTB patients. However, when high-risk factors, such as cavities on chest radiograph and some specific underlying diseases, are encountered in a suspected TB patient, we emphasise that further investigations are required, and empirically treatment for IA might be warranted.

## Data Availability

Data are available upon request from the corresponding authors (WMS and HH).
